# RNA-Dependent RNA Polymerases of Both Virulent and Benign Rabbit Caliciviruses Induce Striking Rearrangement of Golgi Membranes

**DOI:** 10.1371/journal.pone.0169913

**Published:** 2017-01-10

**Authors:** Nadya Urakova, Tanja Strive, Michael Frese

**Affiliations:** 1 CSIRO Health and Biosecurity, Australian Capital Territory, Australia; 2 Invasive Animals Cooperative Research Centre, University of Canberra, Australian Capital Territory, Australia; 3 Health Research Institute, University of Canberra, Australian Capital Territory, Australia; 4 Institute for Applied Ecology, University of Canberra, Australian Capital Territory, Australia; University of Texas Medical Branch at Galveston, UNITED STATES

## Abstract

The extremely pathogenic *Rabbit haemorrhagic disease virus* (RHDV) and the completely benign *Rabbit calicivirus* (RCV) are closely related members of the genus *Lagovirus* (family *Caliciviridae*). The molecular mechanisms that determine the dramatic difference in virulence are unknown, but indirect evidence suggests that different properties of their RNA-dependent RNA polymerases (RdRps) may at least partially be responsible for the contrasting phenotypes. Here we report that the unusual ability of the RHDV RdRp to induce a striking rearrangement of the Golgi network is not specific to RHDV, but a common feature of virulent and benign rabbit caliciviruses alike. Expression of rabbit calicivirus RdRps induced a redistribution of both cis/medial and medial/trans Golgi membrane markers, but not that of an endoplasmic reticulum membrane marker. Inactivating mutations in the conserved GDD motif did not abolish the ability of RHDV RdRp to rearrange the Golgi network, suggesting that polymerase activity and metal co-factors are not required for this function. Finally, we discuss possible implications of RdRp-induced membrane rearrangements on virus replication and host immune responses.

## Introduction

*Rabbit haemorrhagic disease virus* (RHDV) and *Rabbit calicivirus* (RCV) are two closely related viruses in the genus *Lagovirus*, family *Caliciviridae*. Both viruses co-circulate among wild and domestic rabbit populations [1]. RHDV causes an acute lethal infection and 90% of susceptible adult animals usually die from liver failure in less than 72 h [1]. In contrast, RCV causes only a mild, subclinical infection of the small intestine [2, 3].

Despite these significant differences in virulence, both viruses have the same genome organisation and their amino acid (aa) sequences are approximately 87% identical [4]. During lagovirus replication, RNAs of two different types are produced, a 7.4-kb genomic RNA and a 2.1-kb subgenomic RNA; both RNAs are polyadenylated at the 3′ end, covalently linked with the viral genome binding protein (VPg) at the 5′ end and separately packaged into particles [5, 6]. Genomes encode two structural proteins (VP60 and VP10) and seven non-structural proteins (p16, p23, the helicase, p29, VPg, the protease and the RNA-dependent RNA polymerase (RdRp)) [7, 8].

The molecular mechanisms that determine the dramatic difference in the virulence of RHDV and RCV are unknown, largely due to the lack of an effective cell culture system [9]. Indirect evidence suggests that rabbit calicivirus RdRps may at least partially be responsible for the difference in virulence between pathogenic and non-pathogenic rabbit caliviruses, as the two RdRps show different reaction rates (velocities) *in vitro*, with polymerase activity of the RCV RdRp being at least two times higher compared with that of the RHDV enzyme [10]. We and others proposed that a reduced activity of RHDV RdRp may therefore represent a mechanism to increase the fidelity of RNA synthesis [10, 11]. Moreover, a comparative genomic analysis identified an aa site (aa 405) in lagovirus RdRps that distinguished pathogenic RHDV sequences from all known non-pathogenic RCV sequences [12].

We noted previously that the expression of recombinant RHDV RdRp is sufficient to induce a dramatic rearrangement of the Golgi membrane marker giantin in transfected cells [13]. These studies prompted us to explore whether the unusual ability of RHDV RdRp to disrupt the Golgi network is restricted to highly virulent rabbit caliciviruses. To that end, we expressed recombinant RdRps of both the highly pathogenic RHDV and the completely benign RCV in rabbit kidney (RK-13) cells and studied the effects of the protein expression on the Golgi structure. We also analysed the effects of polymerase-inactivating mutations on the ability of RHDV RdRp to disrupt Golgi membranes.

## Methods

### Cells

Rabbit kidney (RK-13) cells (European Collection of Authenticated Cell Culture) were grown in Eagle’s minimal essential medium (EMEM) (Sigma-Aldrich, St. Louis, MO, USA) supplemented with 10% foetal bovine serum (Sigma-Aldrich), 2 mM Glutamax (Gibco, Thermo Fisher Scientific, Waltham, MA USA), 100 μg/ml of streptomycin (Gibco) and 100 units/ml of penicillin (Gibco).

### Plasmids

The construct used for the expression of the C-terminally myc-tagged RHDV RdRp was described previously [13]. The equivalent RCV RdRp construct was generated as follows: RCV RNA was purified from the homogenised small intestine of a rabbit infected with the non-pathogenic calicivirus RCV-A1 [2] using the RNeasy Mini Kit (Qiagen, Hilden, Germany). RCV RNA was reverse transcribed using SuperScript III reverse transcriptase (Life Technologies, Carlsbad, CA, USA). The cDNA encoding RCV RdRp was amplified using Platinum Taq DNA polymerase (Life Technologies) and gene-specific primers containing *Not*I (F, forward; 5’- ATCGTTATAGCGGCCGCCTGCAGTAGCCACCATGA-CTGCAAACTTCTTCTGTG-3’) and *Bam*HI restriction sites (R, reverse; 5’-ATCGTCATCGGATCC-AGATATCCTCCATAACATTCACAAAATCGTC-3’) (restriction sites are underlined). Resulting amplicons were cloned into pcDNA3.1/myc-His(-)C expression vector.

To generate a set of variants of the RHDV RdRp, site-directed mutagenesis of double-stranded plasmid DNA was performed using the following protocol. The respective template plasmid was amplified using Phusion DNA polymerase (Finnzymes, Thermo Fisher Scientific) and specifically designed primers to introduce required substitutions: F:5’-GTATGGCAACGACGGC-3’; R:5’-GTGTAGAATGGGGCGTCC-3’ (GDD→GND); F:5’-GTATGGCGCTGCCGGCGTGTATGC-3’; R:5’-GTGTAGAATGGGGCGTCC-3’ (GDD→ GAA) (substitutions are underlined). The PCR products were treated with *Dpn*I restriction enzyme (New England BioLabs, Ipswich, MA, USA) to remove the template DNA. PCR products of required size were identified using agarose gel electrophoresis and purified using the QIAquick Gel Extraction Kit (Qiagen). Purified PCR products were incubated with T4 polynucleotide kinase (New England BioLabs) at 37°C for 2 h and ligated using the T4 DNA ligase (New England BioLabs) at room temperature for 2 h.

The integrity of the constructs and the presence of required mutations were confirmed by sequencing at the ACRF Biomolecular Resource Facility of the Australian National University (Canberra, ACT, Australia).

To track medial/trans Golgi membranes, rabbit cells were transfected with pECFP-Golgi plasmid (Clontech, Mountain View, CA, USA) to express a fusion protein consisting of cyan fluorescent protein (CFP) fused at its N-terminus to 81 aa of the precursor to the human beta 1,4-galactosyltransferase (1,4-GT).

### Antibodies

Monoclonal mouse anti-myc (M4439), polyclonal rabbit anti-GFP (SAB4301138) antibodies were purchased from Sigma-Aldrich. Polyclonal rabbit anti-giantin (ab24586), polyclonal rabbit anti-calnexin antibodies (ab75801) were purchased from Abcam (Cambridge, UK). Goat anti-mouse IgG AlexaFluor 555 antibodies (A-21424) were purchased from Life Technologies. Goat anti-rabbit IgG DyLight 488 antibodies (GTX76757) were purchased from GeneTex Inc. (Irvine, CA, USA). Anti-calnexin antibodies were used at 1:100 dilution, the remaining antibodies were used at 1:1,000 dilution.

### Immunofluorescence

**Fig 1:**
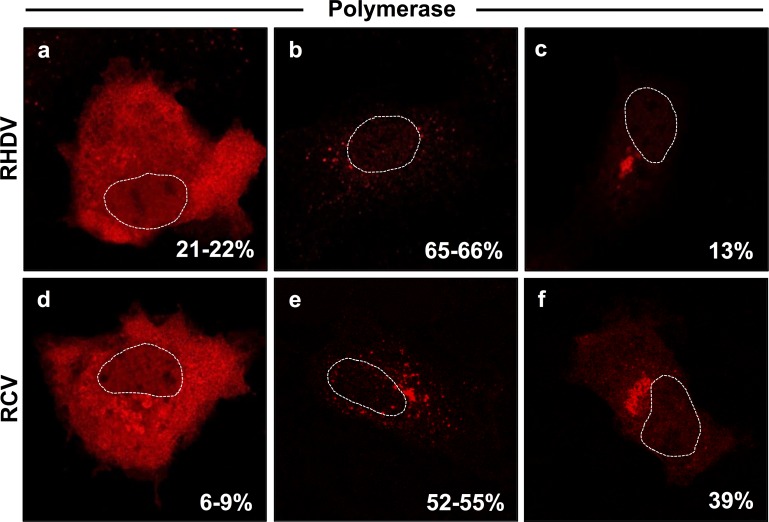
Subcellular localisation of individually expressed recombinant RdRps from different rabbit caliciviruses. RK-13 cells were transiently transfected with expression constructs coding for myc-tagged versions of RHDV RdRp (a–c) or RCV RdRp (d–f). Cells were fixed 24 h after transfections and recombinant proteins were immunostained using myc-specific antibodies. Dotted lines indicate the outline of cell nuclei. Percentages indicate frequencies with which different localisation profiles were found (RHDV and RCV RdRp expression was analysed in 1,002 and 820 transfected cells, respectively, obtained from two independent experiments).

**Fig 2:**
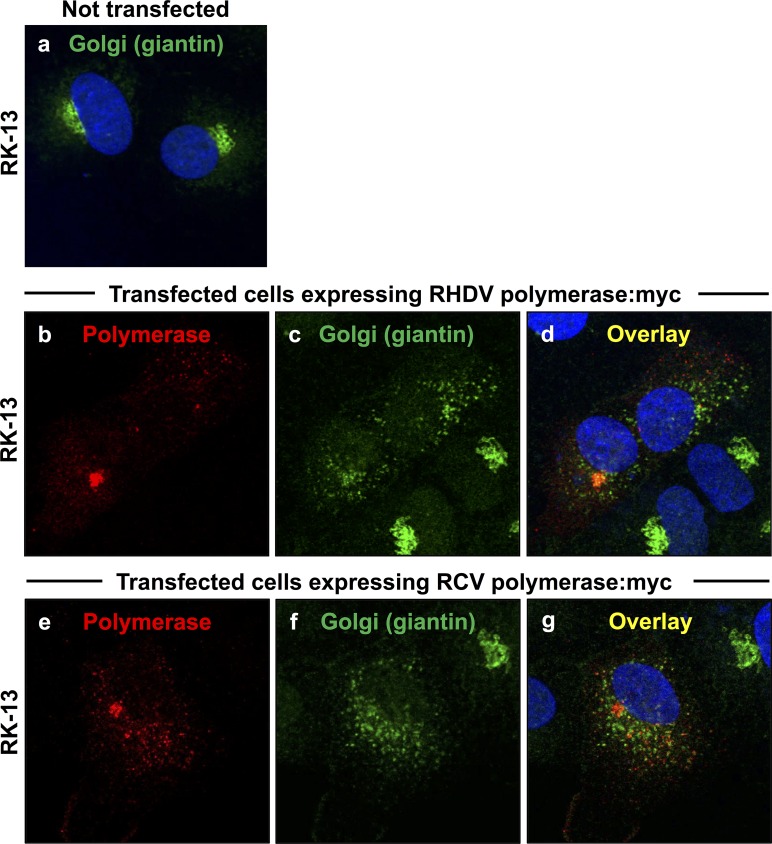
Redistribution of the cis/medial Golgi membrane marker giantin in cells expressing rabbit calicivirus RdRps. RK-13 cells were transiently transfected with expression constructs coding for myc-tagged versions of RHDV RdRp (b–d) or RCV RdRp (e–g). Cells were fixed 24 h after transfections and recombinant proteins were immunostained using anti-myc antibodies (shown in red) and anti-giantin antibodies as a Golgi membrane marker (shown in green). DAPI was used to stain cell nuclei (shown in blue). In cells expressing RHDV or RCV RdRps (b and e, respectively), a striking redistribution of the Golgi membrane marker was observed (c–d and f–g, respectively) as compared with the normal Golgi structure in untransfected control cells (a).

**Fig 3:**
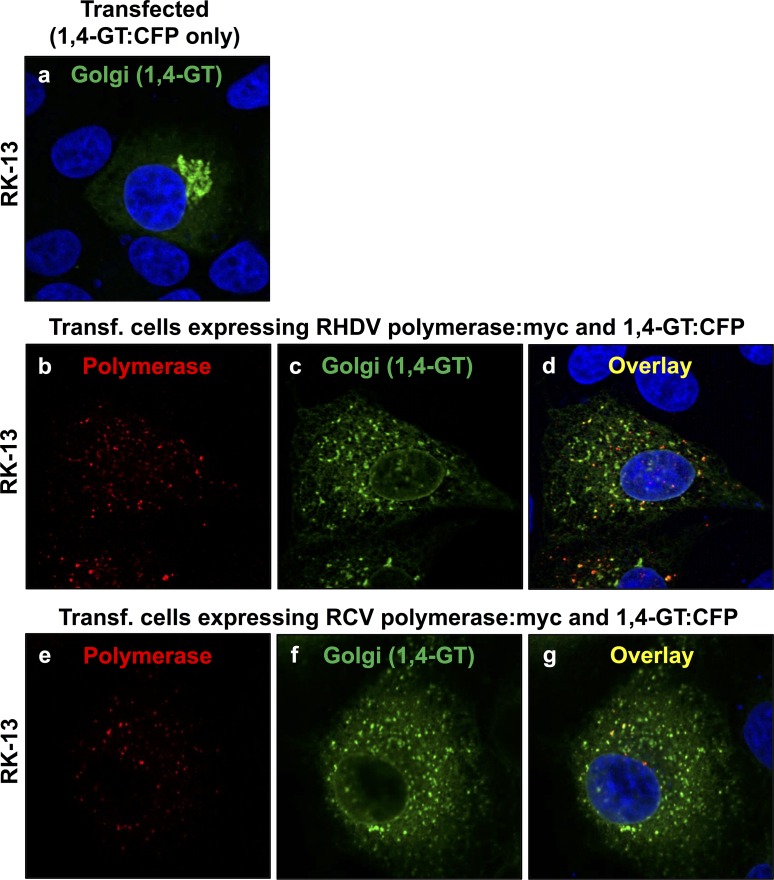
Redistribution of the medial/trans Golgi membrane marker 1,4-GT:CFP in cells expressing rabbit calicivirus RdRps. RK-13 cells were transiently transfected with an expression construct coding for 1,4-GT:CFP (a), co-transfected with constructs coding for 1,4-GT:CFP and RHDV polymerase:myc (b–d) or 1,4-GT:CFP and RCV polymerase:myc (e–g). Cells were fixed 24 h after transfections and recombinant proteins were immunostained using anti-myc antibodies (shown in red) and anti-GFP/CFP antibodies to visualise 1,4-GT:CFP as a Golgi membrane marker (shown in green). DAPI was used to stain cell nuclei (shown in blue). In cells expressing RHDV and RCV RdRps (b and e, respectively), a striking redistribution of the Golgi membrane marker was observed (c–d and f–g, respectively) as compared with the normal Golgi structure in transfected control cells that expressed the 1,4-GT:CFP fusion protein only (a).

**Fig 4:**
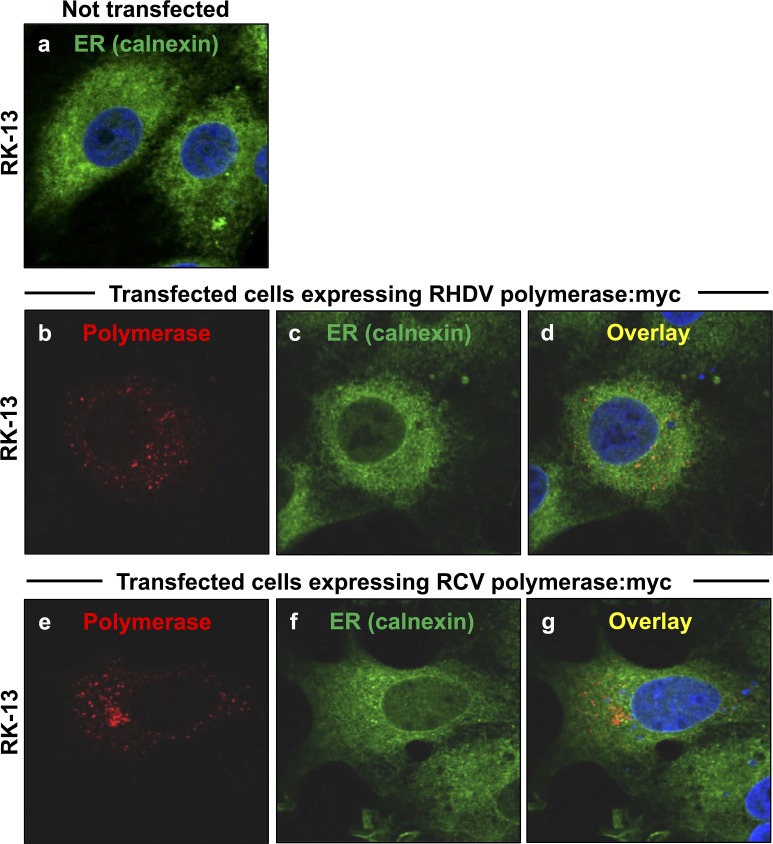
Localisation of the ER membrane marker calnexin in cells expressing rabbit calicivirus RdRps. RK-13 cells were transiently transfected with expression constructs coding for myc-tagged versions of RHDV RdRp (b–d) or RCV RdRp (e–g). Cells were fixed 24 h after transfections and recombinant proteins were immunostained using anti-myc antibodies (shown in red) and anti-calnexin as an ER membrane marker (shown in green). DAPI was used to stain cell nuclei (shown in blue). All cells showed a similar ER staining regardless whether (b–g) or not (a) they expressed RHDV or RCV RdRps.

**Fig 5:**
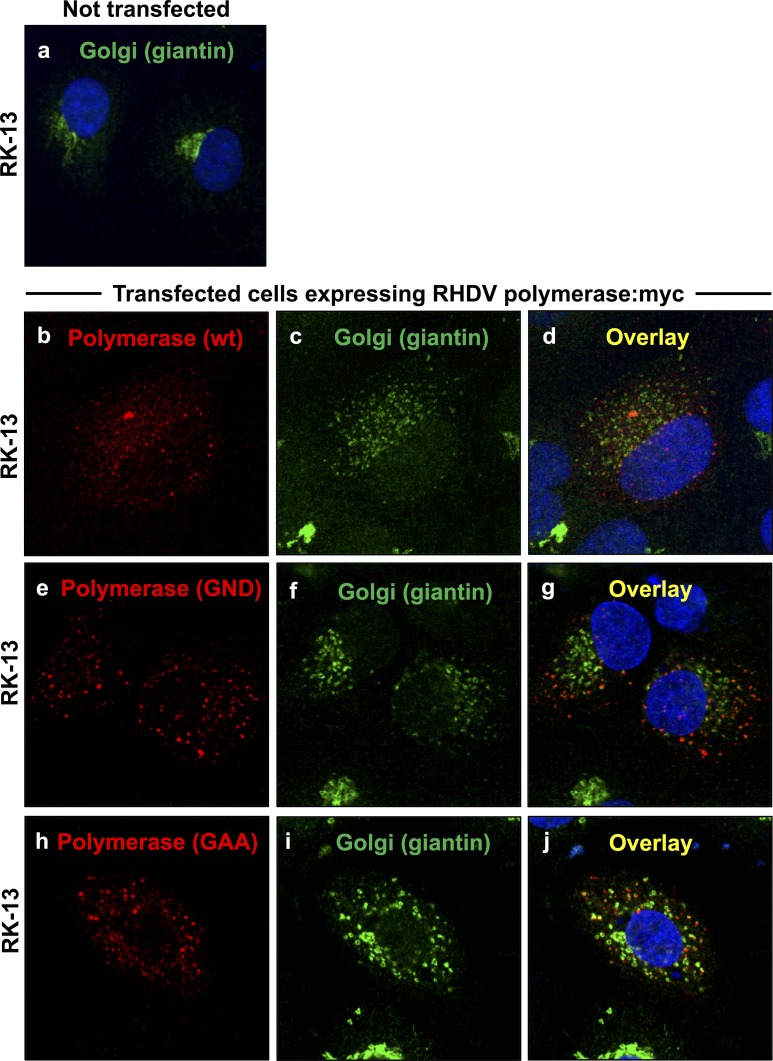
Redistribution of the cis/medial Golgi membrane marker giantin in cells expressing mutant RHDV RdRps. RK-13 cells were transiently transfected with expression constructs coding for a myc-tagged version of the wild-type RHDV RdRp (b–d), or variants containing inactivating mutations in the conserved GDD motif, i.e. GDD→GND (e–g) and GDD→GAA (h–j). Cells were fixed 24 h after transfections and recombinant proteins were immunostained using anti-myc antibodies (shown in red) and anti-giantin antibodies as a Golgi membrane marker (shown in green). DAPI was used to stain cell nuclei (shown in blue). Compared to the normal Golgi structure in untransfected control cells (a), all cells expressing wild-type (b) or variant RdRps (e and h) showed a striking redistribution of giantin (c–d, f–g and i–j).

**Fig 6:**
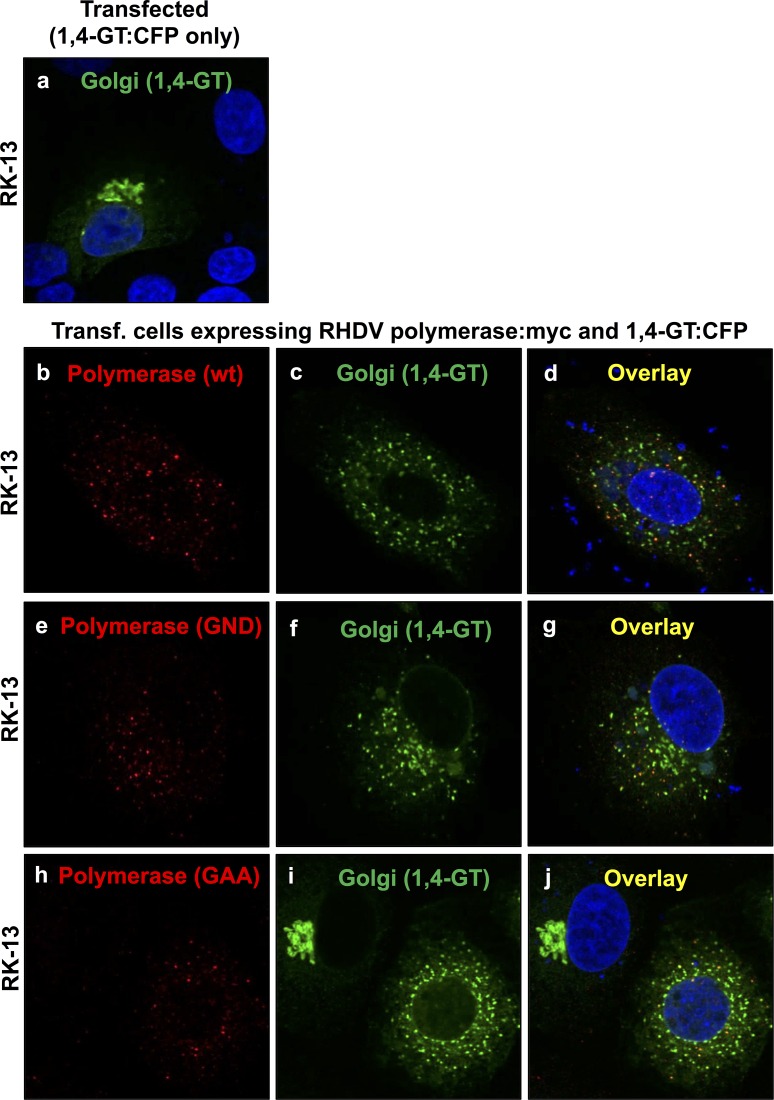
Redistribution of the medial/trans Golgi membrane marker 1,4-GT:CFP in cells expressing mutant RHDV RdRps. RK-13 cells were transiently transfected with an expression construct coding for 1,4-GT:CFP (a), co-transfected with constructs coding for 1,4-GT:CFP and either myc-tagged version of wild-type RHDV RdRp (b–d) or variants containing inactivating mutations in the conserved GDD motif, i.e. GDD→GND (e–g) and GDD→GAA (h–j). Cells were fixed 24 h after transfections and recombinant proteins were immunostained using anti-myc antibodies (shown in red) and anti-GFP/CFP antibodies to visualise 1,4-GT:CFP as a Golgi membrane marker (shown in green). DAPI was used to stain cell nuclei (shown in blue). Compared to the normal Golgi structure in transfected control cells expressing 1,4-GT:CFP only (a), all cells expressing wild-type (b) or variant RdRps (e and h) showed a striking redistribution of 1,4-GT:CFP (c–d, f–g and i–j).

Cells grown on glass coverslips were transfected with expression constructs using Lipofectamine 3000 (Life Technologies). After a 24-h incubation period, cells were fixed with 4% formaldehyde (Polysciences Inc., Warrington, PA, USA) in phosphate-buffered saline (PBS) for 15 min, permeabilised with 0.25% Triton X-100 in PBS for 15 min and incubated for 1 h with blocking solution containing 5% bovine serum albumin (Sigma-Aldrich) in PBS. Primary and secondary antibodies diluted in PBS were incubated for 1 h and 30 min, respectively. Cell nuclei were stained with 4',6-diamidino-2-phenylindole (DAPI) (Sigma-Aldrich). Coverslips were mounted onto glass slides with Fluoromount aqueous mounting medium (Sigma-Aldrich). Images were acquired on a Nikon Ti Eclipse confocal laser-scanning microscope equipped with a 60X objective and analysed using ImageJ software [14]. All fluorescence images shown were chosen to best represent the average staining pattern for a particular experiment. In excess of 1,000 transfected cells were analysed for the experiments shown in Figs 1 and 2, and in excess of 200 transfected cells were analysed for each of the experiments shown in Figs 3–6.

### Cell counting

Transfected cells expressing myc-tagged versions of RHDV and RCV RdRps were stained as described above and examined for a quantitative analysis using a Nikon Eclipse Ti-U fluorescence microscope equipped with a 20X objective. At least 20 view fields from two independent transfections were analysed.

### Amino acid and structure-based alignments

Aa alignments were conducted with BioEdit software (Ibis Biosciences, Carlsbad, CA, USA; http://www.mbio.ncsu.edu/BioEdit/bioedit.html). Structure-based alignments were performed using Discovery Studio software (BIOVIA, San Diego, CA, USA; http://accelrys.com/products/collaborative-science/biovia-discovery-studio/).

## Results

### Subcellular localisation of rabbit calicivirus RdRps

To study the subcellular localisation of rabbit calicivirus RdRps, the coding sequences of RHDV and RCV enzymes were fused with a C-terminal myc-tag and the resulting recombinant proteins were expressed in RK-13 cells. Cells were fixed 24 h after transfection, stained with myc-tag specific antibodies and analysed using indirect immunofluorescence techniques. RdRps of both RHDV and RCV polymerases exhibited similar subcellular localisation profiles (Fig 1). In 21–22% of cells expressing the RHDV RdRp and in 6–9% of cells expressing the RCV RdRp, proteins were diffusely distributed throughout the cytoplasm and the nucleus. In the largest proportion of cells, i.e. 65–66% for RHDV and 52–55% for RCV, proteins accumulated in numerous discrete foci in the cytoplasm. In 13% of cells expressing the RHDV RdRp and in 39% of cells expressing the RCV RdRp, proteins localised in a single and much larger cluster adjacent to the nucleus.

### Rearrangement of Golgi membranes in cells expressing rabbit calicivirus RdRps

A rearrangement of the Golgi network was monitored by analysing the redistribution of two Golgi membrane markers. Giantin-specific antibodies were used to track membranes of cis and medial Golgi compartments [15]. A cyan fluorescent protein (CFP) fused at its N-terminus to 81 aa of the precursor to the human beta 1,4-galactosyltransferase (1,4-GT) was used to follow membranes of medial and trans Golgi compartments [16]. Double immunofluorescence staining with anti-myc and anti-giantin or anti-myc and anti-GFP/CFP antibodies was used to simultaneously stain for recombinant RdRp proteins and various Golgi membranes. At 24 h after transfection, a striking redistribution of both cis/medial and medial/trans Golgi membrane markers was observed in cells expressing RHDV or RCV RdRps (Figs 2 and 3). Although virtually all cells with a detectable lagovirus RdRp expression displayed changes to their Golgi membrane organisation, the particular staining pattern for the rearranged membranes slightly varied from cell to cell. In some cells, the majority of dispersed Golgi membranes was still located close to the nucleus while other cells showed a more even distribution of small vesicles throughout the cytoplasm. In any case, Golgi membrane markers were redistributed irrespective of the polymerase subcellular localisation pattern.

In contrast, no significant changes in the endoplasmic reticulum (ER) compartment were observed when cells were simultaneously stained for ER membranes using antibodies against calnexin [17] and calicivirus RdRps using myc-specific antibodies (Fig 4).

### Polymerase activity is not required for RHDV RdRp to induce rearrangement of Golgi membranes

To test whether polymerase activity is required for the observed Golgi membrane rearrangements, we generated myc-tagged versions of RHDV RdRps with aa substitutions in the conserved GDD motif (i.e. GDD→GND and GDD→GAA). This motif is essential for the coordination of metal ions in the active centre of RdRps, and aa substitutions of the conserved aspartate residues in the GDD motif completely eliminate or greatly affect the enzymatic activity of these polymerases [18–20]. Variant RHDV RdRps were generated by site-directed mutagenesis, recombinant myc-tagged proteins were expressed in transfected RK-13 cells and the effect of the recombinant proteins on Golgi membranes was studied. Neither of these aa substitutions changed the subcellular localisation profile of the RdRp (data not shown) or abolished its ability to induce a redistribution of cis/medial and medial/trans Golgi membrane markers (Figs 5 and 6, respectively). As described for the expression of lagovirus RdRps with an intact GDD motif, the expression of variant proteins changed the Golgi membrane organisation in nearly all cells. Furthermore, we did not find that expression of any of the mutants caused a significant change to staining pattern of the dispersed Golgi membrane vesicles (data not shown).

## Discussion

We found that expression of both RHDV and RCV RdRps induced a significant redistribution of Golgi membrane markers in transfected rabbit cells. The results extend previous findings [13] and indicate that the unusual ability of rabbit calicivirus RdRps to rearrange Golgi membranes is a common characteristic of all rabbit caliciviruses, and not a specific attribute of highly virulent strains.

Since replication of single-stranded positive sense RNA viruses occurs on modified intracellular host membranes [21–23], we propose that lagovirus RdRps may play a key role in the biogenesis of the membranous organelle-like structures required for viral replication. We demonstrated previously that Golgi membrane redistribution occurs in transfected cells that express the RHDV RdRp alone or in the context of the entire RHDV polyprotein [13], suggesting that lagoviruses, similar to other caliciviruses and picornaviruses [24, 25], may indeed recruit cellular membranes of the secretory pathway for their replication machinery. However, it should be noted that the exact source and structure of membranous compartments forming in RHDV- and RCV-infected cells is unknown and should be the subject of future investigations (so far, no cell culture system has been established for any lagovirus).

**Fig 7:**
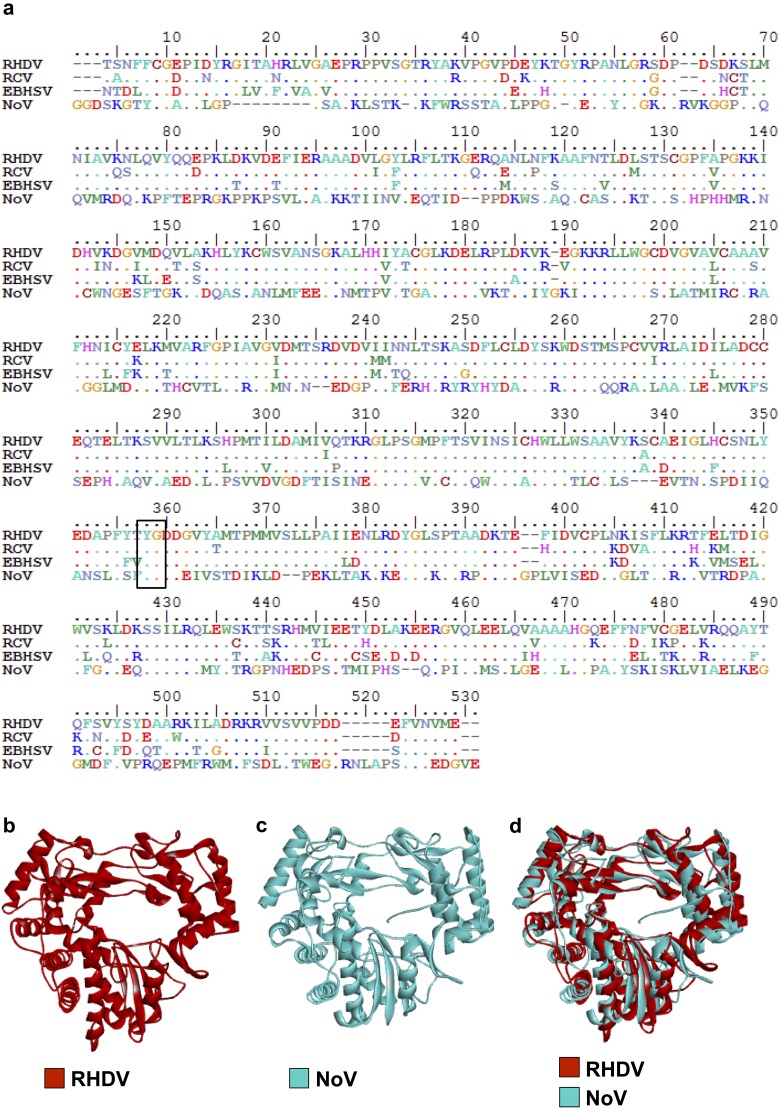
Comparison of calicivirus RdRps. (a) RdRp aa sequence alignment of RHDV Czech strain V351 (GenBank accession number KF594473.1), RCV-A1 (GenBank accession number EU871528.1), *European brown hare syndrome virus* (EBHSV) (GenBank accession number NC_002615.1) and NoV (GenBank accession number AJ583672.2). The conserved GDD motif is marked with a black box. (b, c) Crystal structure of RHDV (Protein Data Bank ID 1KHW) and NoV (Protein Data Bank ID 1SH2) RdRps, respectively. (d) Superimposed structures of RHDV (shown in red) and NoV (shown in cyan) RdRps demonstrating the high degree of structural similarity between calicivirus polymerases.

Our discovery that the expression of lagovirus RdRps induces the rearrangement of Golgi membranes is unexpected, because most of the mechanisms of virus-induced membrane reorganisation described to date are attributed to viral proteins other than RdRps. Although polymerase-dependent membrane rearrangements were described for members of *Nodaviridae* family, such as *Flock house virus* and *Nodamura virus*, it should be noted that the RdRps of these viruses are twice as large as their RHDV and RCV counterparts and that they carry distinct functional domains in addition to the core polymerase structure present in all RdRps [26, 27]. Rabbit calicivirus RdRps, on the other hand do not have any obvious domains or motifs that could be easily linked to the observed Golgi membrane rearrangements. Moreover, the RdRp of RHDV (RdRps of other lagoviruses have not yet been crystallised) has a remarkable structural homology with RdRps of other caliciviruses such as human norovirus (NoV) (see Fig 7B–7D and S1–S3 Movies) [28, 29]. Nevertheless, no direct interactions with membranes or effects on the host cell membrane architecture have so far been reported for RdRps of caliciviruses other than RHDV or RCV. For example, no such interactions were observed in cells expressing the RdRp of murine norovirus [30].

Since the Golgi apparatus plays an important role in the processing of proteins destined for secretion, it is tempting to speculate that the fragmentation of the Golgi network may block the secretory pathway. This would reduce the expression of histocompatibility complex proteins on the plasma membrane and the secretion of proinflammatory and/or antiviral cytokines, e.g. interferons, as it was shown for members of the picornavirus family [31–33]. An inhibition of protein secretion may therefore alter host immune responses. Further studies will reveal how rabbit calicivirus RdRps affect the intracellular transport of host proteins through the remains of the Golgi apparatus, and whether infected cells can evade innate and/or adaptive immune responses. Prompted by a request from one of our reviewers, we performed a small series of experiments in which we used the *Gaussia* luciferase reporter system [34] to analyse the functionality of the Golgi apparatus. We found that the expression of RdRps did not reduce the luciferase activity levels in the supernatant of transfected cells, but we consistently observed higher intracellular luciferase levels in cultures that expressed lagovirus RdRps, especially in those that expressed the RHDV RdRp (data not shown). The biological significance of these preliminary findings is not clear and further experiments are required. We also tested whether the polymerase activity of rabbit calicivirus RdRps is required for the disruption of the Golgi apparatus. In contrast to the replication protein A of *Flock house nodavirus*, for which polymerase activity was found to be essential to induce spherule formation on the outer mitochondrial membrane [26], polymerase inactivating aa substitutions in the GDD motif of the RHDV RdRp did not significantly change the ability of the protein to disrupt the Golgi network (Figs 5 and 6). The result suggests that metal cofactors and ongoing RNA synthesis are not required for RdRps of rabbit caliciviruses to rearrange intracellular membranes.

Previous results indicate that the presence of different subcellular localisation profiles for RdRp is not caused by major degradation or processing events of the recombinant protein in transfected cells [13]. It is, however, possible that RdRps undergo other post-translational modifications, such as palmitoylation, which may affect the subcellular localisation of the protein.

Interestingly, the majority of picornavirus proteins that inhibit the secretory pathway, such as 2B, 2BC, 2C and 3A, have membrane-binding motifs and associate with membrane vesicles [31]. We show here that both RHDV and RCV RdRps have a rather unusual subcellular localisation: in a large proportion of transfected cells they accumulate in distinct, but as yet undefined subcellular structures (Fig 1). We did not observe a consistent co-localisation between RdRp and Golgi membranes. Whether there is a partial or temporal co-localisation is presently unknown. Considering the complex subcellular localisation profile of rabbit calicivirus RdRps and their ability to rearrange Golgi network, we speculate that rabbit calicivirus RdRps may associate with subcellular vesicles either directly or indirectly via membrane-associated host proteins. It is further possible that only a proportion of all RdRp proteins engage with Golgi membranes or membrane bound proteins and that this interaction depends on protein modifications. However, the putative mechanism of these interactions remains elusive.

Although the aa sequences of viral RdRps are highly conserved only among closely related viruses and within essential functional motifs [20, 29], calicivirus RdRps show a remarkable degree of structural similarity (Fig 7 and S1–S3 Movies). Due to this structural homology, knowledge acquired from studying a specific polymerase is usually broadly applied to other related polymerases. Compared to RdRps from other caliciviruses (e.g. NoV), the RHDV enzyme does not have any obvious additional structures. This suggests that either rabbit calicivirus RdRps have evolved existing protein structures/motifs to perform additional functions or that other calicivirus RdRps are also able to execute similar activities. If the latter case is true, these functions have not been demonstrated in the literature so far.

## Conclusions

This is the first study that describes the subcellular localisation of RCV RdRp and the effect of its expression on the Golgi network. Our results indicate that the rather unusual subcellular localisation of rabbit calicivirus RdRps and their ability to rearrange Golgi membranes are common characteristics of this virus taxon rather than attributes of highly virulent strains. We further show that polymerase activity of rabbit calicivirus RdRps is not essential for the disintegration of the Golgi apparatus.

## Supporting Information

S1 Movie RHDV RdRp crystal structure. This video clip shows the RHDV RdRp crystal structure in 3D (Protein Data Bank ID 1KHW). Amino acids that are common to RHDV and RCV are shown in red, and amino acids that differ between the two viruses are highlighted in yellow.
(MP4)

S2 Movie NoV RdRp crystal structure. This video clip shows the NoV RdRp crystal structure in 3D (Protein Data Bank ID 1SH2). Amino acids that are common to NoV and RHDV are shown in cyan, and amino acids that differ between the two viruses are highlighted in yellow.
(MP4)

S3 Movie RHDV and NoV RdRp crystal structure. This video clip shows the superimposed crystal structures of RHDV (red) and NoV (cyan) in 3D.
(MP4)
